# Professional burnout of nurses and the level of rationing of nursing care: an observational preliminary study

**DOI:** 10.1186/s12912-024-01940-x

**Published:** 2024-04-24

**Authors:** Patrycja Marczak, Dorota Milecka

**Affiliations:** 1 Medical Institute, State University of Applied Sciences in Głogów, Piotra Skargi 5 Street, 67-200 Głogów, Poland; 2 Medical Institute, State University of Applied Sciences in Głogów, Piotra Skargi 5 Street, 67-200 Głogów, Poland

**Keywords:** Nurses, Professional burnout, Rationing of nursing care, BERNCA-R

## Abstract

**Background:**

Nurses are one of the professional groups most exposed to experiencing professional burnout. Professional burnout has a negative impact on the quality of nursing care, including causing care rationing. Therefore, it is very important to understand the determinants of both professional burnout and care rationing, as well as their mutual relationships. The aim of the study was to understand the impact of professional burnout among nurses on the level of rationing of nursing care.

**Methods:**

The study was conducted among 100 nurses at the Głogów County Hospital (Poland) from November 14, 2011, to November 18, 2022. The following Polish version of the standardized research tools were utilized: the Basel Extent of Rationing of Nursing Care– Revised (BERNCA-R) questionnaire and the Oldenburg Burnout Inventory (OLBI). Additionally, a survey designed by the authors was employed.

**Results:**

The BERNCA-R significantly correlates (*p* < 0.05) and positively (*r* > 0) with OLBI (disengagement), resulting in a higher degree of care rationing. The average overall BERNCA-R score was 1.56 points (SD = 0.62), indicating that the frequency of care rationing among respondents ranged from “never” to “rarely.” Among participants in the OLBI questionnaire, 63% of respondents had a moderate level of work exhaustion, 36% had a high level of work exhaustion, and 1% had a low level of work exhaustion. In turn, 58% of respondents had a moderate level of disengagement, 38% had a high level of disengagement, and 4% had a low level of disengagement. Moreover, a statistically significant association with the BERNCA-R score concerning the workplace (ward) and participation in training on preventing professional burnout was shown.

**Conclusions:**

The rationing of nursing care was found to be at a low level. The higher the level of disengagement, the greater the level of care rationing was observed. In conservative units, nurses demonstrated a higher level of care rationing. Nurses’ expectations regarding the reduction of professional burnout include, among other things, higher remuneration, an increase in the number of staff, and an improvement in the work atmosphere.

## Background

Rationing of nursing care, as a concept, was first defined in 2006. It refers to the omission of entire, partial, or individual aspects of care [1]. Rationing of nursing care occurs when the available resources are insufficient to provide the required care to each patient. Among the factors contributing to this phenomenon are: staff reductions, new technologies that increase the demand for care, as well as new therapies and treatment options, and the level of patient knowledge [2]. Kalisch [1] identified 9 nursing activities that are most commonly omitted: repositioning, hygiene care, feeding, mobilizing patients, education, emotional support, documentation, discharge planning, and supervision. Rationing of nursing care is the delay or complete omission of required nursing activities. Rationing occurs when a nurse does not have sufficient resources to perform designated tasks. As a result, she is forced to decide which tasks will be postponed or entirely omitted [3]. According to Schubert [4], the concept of rationing of nursing care assumes that all actions performed by the nurse are equally important, facilitating the achievement of desired goals and actions expected by patients. These actions include diagnostic activities, prevention, rehabilitation, emotional support, and therapy. The nurse conducts a clinical assessment and presents nursing diagnoses, so difficulties in achieving the accepted care goals lead to the rationing of nursing care [5].

Problem of rationing of nursing care is clearly noticeable worldwide and poses a threat to patient safety. The Supreme Council of Nurses and Midwives in Poland [6] prepared a report in 2017 showing the employed and registered nurses and midwives from 2016 to 2030. The results indicate an increase in the number of registered nurses and midwives, but with pension rights, which means that staffing issues will deepen even further in the future.

One of the factors influencing the rationing of nursing care is professional burnout, stemming from emotional exhaustion, low job and life satisfaction, as well as fatigue and perceived stress [7]. A low level of resources in the workplace is one of the more significant causes of professional burnout, leading to a reduction in nurses’ engagement in performing their duties. The increase in the number of employees affected by professional burnout results in an increase in employee turnover. Consequently, by filling staffing gaps often with less qualified personnel, there is a decrease in the quality of care, including patient safety [7, 8].

Due to the nature of their work, nurses are one of the leading professional groups most susceptible to professional burnout. The mechanism behind professional burnout is attributed to chronic stress [9]. According to nurses, the main sources of stress include: the pressure of being responsible for patients’ health and life [10, 11], unsatisfactory financial gratification [10, 12] inadequately equipped workstations, insufficient staffing, lack of support from superiors, demanding families of the patients, which, according to respondents, contributes to a lack of respect for their work [10], as well as fear of making mistakes and dealing with death [11].

Professional burnout leads to neglect in nursing care, resulting in more frequent occurrences of medical errors and adverse events. Therefore, preventive measures against professional burnout and the rationing of nursing care are extremely important. Considering the limited evidence on the association between professional burnout and the rationing of nursing care in the literature, the main objective of the conducted research was to determine the impact of nurses’ professional burnout on the level of rationing of nursing care.

## Methods

### Ethical considerations

The study was approved by the local Bioethics Committee of the Medical Institute at the State University of Applied Sciences in Głogów, Poland (no. 43/2022). The study adhered to the principles of the Helsinki Declaration and Good Clinical Practice, and it followed the STROBE guidelines for comprehensive and transparent reporting of observational studies.

### Study participants

The study was conducted from November 14, 2021, to November 18, 2022, at the Głogów County Hospital. This hospital was chosen as the setting for our study due to several reasons. The hospital serves a diverse patient population, providing a broad representation of cases and conditions encountered in nursing practice. Moreover, the Głogów County Hospital has a well-established nursing staff with varying levels of experience and expertise, offering insights into different aspects of nursing care and potential factors contributing to professional burnout. And finally, the hospital administration expressed interest in participating in research aimed at improving nursing practice and staff well-being.

The study included a group of 110 nurses directly involved in patient care. One hundred questionnaires were returned, representing a response rate of 90.9%. The study ensured anonymity by deliberately excluding personal identifiers such as names and contact information from the survey forms. This approach aimed to prevent any potential identification of respondents throughout the data collection process. The questionnaires were administered during designated times, ensuring participation from a representative sample of nurses across different shifts and units within the hospital. All data collection procedures adhered to ethical guidelines and were conducted with full consent and confidentiality assured to participants.

### Selection criteria

Participants eligible for inclusion in the study were required to be practicing nurses actively engaged in direct patient care responsibilities, such as administering medications and performing assessments. Additionally, participants had to provide informed consent to participate in the survey, indicating their willingness to share their experiences and opinions. Exclusion criteria encompassed individuals not directly involved in patient care, such as administrative staff or educators, as well as those holding managerial or supervisory roles within the nursing department. Furthermore, individuals who declined to participate in the survey were excluded from the study population.

### Research tools

The Basel Extent of Rationing of Nursing Care Revised (BERNCA-R) questionnaire was developed by Schubert et al. [13] to determine the level of rationing of nursing care. The tool has a Polish language version of which the Cronbach’s alpha coefficient is 0.96 [14]. The questionnaire consists of 32 questions concerning situations in which rationing of nursing care may occur. The questions relate to activities related to patient care, including addressing the patient’s biopsychosocial problems, providing educational and emotional support, monitoring the patient’s condition, implementing medical orders, and conducting the nursing care process along with documenting the provided nursing care. Each question is assessed on a scale: no such need (0 points), never (1 point), rarely (2 points), sometimes (3 points), often (4 points). Respondents assessed how often in the last 7 working days they were unable to perform the activities listed in the questionnaire. The measurement is the sum of points, where a higher score indicates a higher level of rationing of nursing care.

The Oldenburg Burnout Inventory (OLBI) was developed by Demerouti et al. [15]. The tool was designed to measure burnout among different professional groups. The questionnaire allows the measurement of two dimensions: exhaustion and disengagement from work, consisting of 16 statements, eight for each of the two subscales [16]. There is a Polish adaptation of the OLBI of which the Cronbach’s alpha coefficient is 0.80 for exhaustion and 0.76 for disengagement from work [17], in which each subscale contains four positively and four negatively formulated items. Consequently, two ends of the measured dimensions are assessed: exhaustion-vigor and cynicism-dedication. Respondents mark one answer on a 4-point scale (where 1 means strongly agree and 4 means strongly disagree). The questionnaire does not have a neutral option. Scores are summed for negatively formulated questions, and then the average is calculated for each category/dimension. Higher scores indicate a greater intensity of the respective phenomenon.

The custom questionnaire consists of 20 questions (19 closed-ended, 1 open-ended, “Write what your employer can do– what is important for you at work to be satisfied with your job?“). The questionnaire comprised three parts. The first part pertained to sociodemographic characteristics such as age, gender, marital status, current education, and postgraduate education. The second part focused on job-related characteristics associated with the position of a nurse: department, length of service, number of job positions (full-time equivalents), number of overtime hours, working system, average number of patients under the care of a nurse during one shift, and participation in training on preventing professional burnout. The third part addressed the expectations of nurses regarding preventing professional burnout. Participants were asked questions such as: What can contribute to reducing professional burnout?; How many patients should one nurse be responsible for?; What preventive activities for professional burnout should be organized?; Are financial rewards granted?; Do employers commend well-performed work?

### Statistical analysis

Analyses of quantitative variables (expressed in numbers) were conducted by calculating the mean, standard deviation, median, and quartiles. Analysis of qualitative variables (not expressed in numbers) was carried out by calculating the count and percentage of occurrences for each value. Analyzed variables did not follow a normal distribution, which was confirmed by the Shapiro-Wilk test. The comparison of quantitative variable values in two groups was performed using the Mann-Whitney U test. The comparison of quantitative variable values in three or more groups was done using the Kruskal-Wallis test. After detecting statistically significant differences, post-hoc analysis was performed using the Dunn test to identify groups that differed significantly. Correlations between quantitative variables were analyzed using the Spearman correlation coefficient. A significance level of 0.05 was adopted for the analysis. Thus, all *p*-values below 0.05 were interpreted as indicating significant relationships. The analysis was conducted using the R software, version 4.2.2 [18].

## Results

The group of participants in the study consisted of 100 nurses (Table 1). The largest group of participants were women– 87, while men were only– 13. The majority of participants in the study were in the age range of 20–30 years– 31 individuals. The most numerous group of respondents were individuals in a formal relationship– 49 people. The largest educational group, totaling 48 people, had a bachelor’s degree in nursing. Regarding postgraduate education, it was a multiple-choice question, and the respondents most frequently participated in qualification courses– 40 individuals, followed by specialized courses– 35 individuals, and nursing specialization– 29 individuals.

**Table 1:** Sociodemographic characteristics of the study group

Variable		n	%
Age	20–30 years old	31	31%
	31–40 years old	29	29%
	41–50 years	28	28%
	51–60 years	9	9%
	61 years and over	3	3%
Sex	Female	87	87%
	Male	3	13%
Education	Medical high school	12	12%
	Medical school	8	8%
	Bachelor of nursing	48	48%
	Master of Science in Nursing	30	30%
	Other	2	2%
Postgraduate education	Specialization in nursing	29	29%
	Specialization in organization and management	10	10%
	Qualification course	40	40%
	Specialized course	35	35%
	Further training course	12	12%
Marital status	Single	23	23%
	Formal relationship	49	49%
	Partnership relationship	20	20%
	Widowed	5	5%
	Divorced	3	3%

***Notes:*** *The percentages do not add up to 100 because it was a multiple-choice question

The department with the highest number of employees is the conservative ward– 31 individuals, and a similar number of people work in the surgical ward– 30 individuals. The most numerous group in terms of work experience were individuals with 0–5 years of experience– 45 individuals. The largest number of respondents declared working in one place– 45 individuals. The shift work system is the most frequently declared response– 84 individuals. The largest number of respondents works an additional 25 h per month– 32 individuals. The majority of respondents stated that they are responsible for 11–15 patients during a shift– 39 individuals, followed by 6–10 patients– 34 individuals. More than half of the study participants– 55 individuals– declared no participation in training on preventing professional burnout (Table 2).

**Table 2:** Characteristics of the study group by character of work

Variable		n	%
Ward	Conservative	31	31%
	Surgical	30	30%
	Pediatric	12	12%
	Pediatric surgery	5	5%
	ICU	20	20%
	Other	2	2%
Seniority in the profession	0–5 years	45	45%
	6–10 years	19	19%
	11–15 years	16	16%
	16–20 years	9	9%
	21 years and over	11	11%
Number of jobs	1 job	45	45%
	2 jobs	34	34%
	3 and more jobs	21	21%
Monthly number of overtime hours	More than 100 h	9	9%
	Approximately 75 h	19	19%
	Approx. 50 h	27	27%
	Approximately 25 h	32	32%
	Not at all	13	13%
System of work	Single shift system	5	5%
	Shift system	84	84%
	Mixed system	11	11%
Number of patients under care while on duty	1–5 patients	12	12%
	6–10 patients	34	34%
	11–15 patients	39	39%
	16–25 patients	10	10%
	26–35 patients	4	4%
	36 or more patients	1	1%
Participation in training on preventing professional burnout	Yes	24	24%
	No	55	55%
	Do not remember	21	21%

Table 3 presents the results of the analysis of nurses’ expectations regarding the reduction of professional burnout. In response to the question “In your opinion, what could contribute to reducing professional burnout?“, the highest number of individuals selected the answer “Employing more nurses”– 61 people. The second most frequently chosen response was “Higher salary”– 49 people. The next most frequently selected responses, ranging from 39 to 38%, were “Better work atmosphere” and “Greater access to auxiliary staff.” Other responses received 29% and below 29% of participants’ answers in the study. The fewest respondents selected the answer “Greater access to training on preventing professional burnout” and “Greater support from nursing management”– 9 people each. According to the majority of respondents, one nurse should be responsible for 1–5 patients– 92 individuals, 6–10 patients– 6 individuals, and 11–15 patients– 2 individuals. Regarding activities related to preventing professional burnout, the highest number of individuals declared a willingness to participate in mindfulness-based stress reduction training– 33 people. In response to the question about receiving a financial reward, the most frequently chosen answer was “Never”– 44 people. The highest number of respondents in the question “Have you ever received praise for your work?” selected the answer “Several times”– 32 people (32%).

**Table 3:** Characteristics of the study group due to activities in reducing burnout levels

Variable		n	% *
What can contribute to reducing professional burnout?	Hiring more nurses	61	61%
	Greater access to support staff	38	38%
	Increased access to equipment for moving, lifting, transporting, and mobilizing patients	25	25%
	Enhanced access to training in preventing burnout	9	9%
	Greater availability for professional development	16	16%
	Better work atmosphere	39	39%
	Increased opportunity to make decisions regarding patient care	14	14%
	Improved communication within the team	29	29%
	Enhanced teamwork	22	22%
	Better work organization	24	24%
	Reduced bureaucracy	17	17%
	Increased professional autonomy	28	28%
	Higher salary	49	49%
	Patient assignment to specific nurses	21	21%
	Greater support from nursing management	9	9%
	Delegation of tasks that are not part of the nurse’s responsibilities	21	21%
	Improved workplace equipment	18	18%
How many patients should one nurse be responsible for?	1–5 patients	92	92%
	6–10 patients	6	6%
	11–15 patients	2	2%
What preventive activities for professional burnout should be organized?	Supervision	5	5%
	Balint group	12	12%
	Assertiveness training	25	25%
	Positive thinking training	19	19%
	Mindfulness-based stress reduction training	33	33%
	Yoga classes	13	13%
Are financial rewards granted?	Never	44	44%
	I don’t remember	20	20%
	Once	20	20%
	Several times	13	13%
	Several times	3	3%
Do employers commend well-performed work?	Never	25	25%
	I don’t remember	30	30%
	Once	9	9%
	Several times	32	32%
	Several times	4	4%
What model of patient care do you prefer?	Model 1	40	40%
	Model 2	17	17%
	Model 3	32	32%
	Model 4	11	11%

***Notes:*** The percentages do not add up to 100 because it was a multiple-choice question.

***Legend***: Model 1: Care based on the nursing process method, with patient allocation to nurses, including patient assessment, identification of nursing diagnoses, care planning, implementation, and evaluation of nursing interventions. Model 2: Care based on the traditional model– nursing subservient to the doctor’s decisions, where the nurse’s work mainly involves carrying out medical orders. Model 3: Care based on the traditional model– nursing subservient to the functional specialization of the nurse, i.e., dividing nurses into groups based on tasks, for example, one nurse administers intravenous infusions, another administers oral medications, etc. Model 4: Care based on the traditional model– a combination of nursing subservient to the doctor’s decisions with nursing subservient to the functional specialization of the nurse.

The most frequently chosen answer to the question “What patient care model do you prefer?” was (Model 1) the care method through the nursing process, involving the allocation of patients to nurses and the recognition of the patient’s condition, identification of nursing diagnoses, planning of patient care, implementation, and evaluation of nursing actions– 40 individuals. According to respondents’ answers to the question “What can the employer do to make you satisfied with your job?“, it can be inferred that the most expected action from the employer is salary increases and the awarding of recognition bonuses. An interesting suggestion from the respondents was also the organization of meetings with superiors to listen to the team’s expectations, grievances, and problems. Respondents expressed a desire to participate in courses and training organized and paid for by the employer. Some individuals reported the issue of outdated medical equipment. Nurses also showed a willingness to participate in team-building meetings to better get to know each other and enhance understanding among them (given the significant age difference among employees) (Table 3).

As much as 63% of participants experienced a moderate level of work exhaustion, 36% had a high level, and 1% of respondents had a low level. Regarding the level of job disengagement, 58% had a moderate level, 38% had a high level, and 4% had a low level (Table 4).

**Table 4:** The results of the OLBI and BERNCA-R questionnaires analysis

Questionnaire		Mean	SD
OLBI: Exhaustion	Overall result	2.58	0.35
	Interpretation	**N**	**%**
	Low level (1-1.90 pts.)	1	1%
	Moderate level (1.91–2.74 pts.)	63	63%
	High level (2.75–4 pts.)	36	36%
OLBI: Disengagement	Interpretation	**Mean**	**SD**
	Overall result	2.59	0.37
	Interpretation	**N**	**%**
	Low level (1-1.88 pts.)	4	4%
	Moderate level (1.89–2.71 pts.)	58	58%
	High level (2.72-4 pts.)	38	38%
BERNCA-R	Interpretation	**Mean**	**SD**
	Overall result	1.56	0.62

***Abbreviations***: OLBI: the Oldenburg Burnout Inventory, BERNCA-R: the Basel Extent of Rationing of Nursing Care Revised

Table 5 presents the distribution of responses to individual questions in the BERNCA-R questionnaire. The most frequently rationed (highest average) were: assisting patients with limited/difficult mobility or immobilized in movement (question 7), observing confused patients, requiring them to be immobilized (question 20), activities related to oral hygiene of the patient (question 4), activities related to the patient’s tooth hygiene (question 5), and changing the position of patients with limited/difficult mobility or immobilized (question 8).

**Table 5:** Responses to individual questions on the BERNCA-R questionnaire

Question	There was no need (0)	Never (1)	Rarely (2)	Sometimes (3)	Often (4)	No answer	Mean
1	10%	44%	27%	13%	6%	0%	1.61
2	13%	43%	28%	12%	4%	0%	1.51
3	10%	51%	22%	12%	5%	0%	1.51
4	7%	45%	26%	17%	5%	0%	1.68
5	9%	42%	26%	18%	5%	0%	1.68
6	10%	47%	25%	15%	3%	0%	1.54
7	7%	37%	32%	17%	7%	0%	1.8
8	7%	37%	38%	17%	1%	0%	1.68
9	5%	43%	35%	14%	3%	0%	1.67
10	6%	43%	31%	17%	3%	0%	1.68
11	5%	45%	31%	19%	0%	0%	1.64
12	7%	46%	26%	20%	1%	0%	1.62
13	19%	37%	21%	22%	1%	0%	1.49
14	27%	35%	22%	16%	0%	0%	1.27
15	17%	33%	27%	19%	4%	0%	1.6
16	15%	50%	23%	11%	1%	0%	1.33
17	11%	50%	25%	11%	3%	0%	1.45
18	7%	44%	34%	12%	3%	0%	1.6
19	4%	49%	31%	11%	5%	0%	1.64
20	10%	35%	30%	21%	4%	0%	1.74
21	11%	36%	31%	19%	3%	0%	1.67
22	8%	46%	29%	14%	3%	0%	1.58
23	8%	52%	27%	12%	1%	0%	1.46
24	5%	60%	23%	9%	3%	0%	1.45
25	6%	59%	24%	9%	2%	0%	1.42
26	12%	53%	18%	15%	2%	0%	1.42
27	12%	55%	20%	10%	3%	0%	1.37
28	6%	62%	21%	10%	1%	0%	1.38
29	5%	47%	35%	12%	1%	0%	1.57
30	6%	47%	33%	11%	3%	0%	1.58
31	4%	47%	32%	16%	1%	0%	1.63
32	5%	50%	25%	18%	2%	0%	1.62

In our study, no statistically significant relationships were found indicating the influence of sociodemographic characteristics such as age, gender, undergraduate education, postgraduate education, and marital status on the level of rationing of nursing care (Table 6). In our study, statistically significant dependencies were found with the BERNCA result concerning the workplace (ward) (p˂0.05) and participation in training on preventing burnout (p˂0.05). The frequency of care rationing was significantly higher in conservative than in surgical, pediatric ward, and ICU wards. Additionally, the frequency of care rationing was significantly higher among those who couldn’t recall whether they participated in burnout prevention training compared to those who did not participate. However, no significant dependencies were observed concerning work experience, the number of job positions, monthly overtime hours, work schedule, and the number of patients under the nurse’s care during a shift (Table 6).

**Table 6:** Effect of selected variables on nursing care rationing

Variable		BERNCA-R [points]									
		N	*X*	SD	Me	Min	Max	Q1	Q3	*p*	
Age	20–30 years old	31	1.48	0.47	1.41	0.47	2.22	1.19	1.83	*p* = 0.666*	
	31–40 years old	29	1.64	0.62	1.69	0.59	3.19	1.12	2.00		
	41–50 years	28	1.59	0.66	1.59	0.34	3.12	1.17	1.95		
	51 years and over	12	1.49	0.89	1.23	0.38	3.22	0.87	1.88		
Sex	Female	87	1.59	0.63	1.62	0.34	3.22	1.16	1.98	*p* = 0.282**	
	Male	13	1.37	0.54	1.38	0.59	2.25	1.00	1.78		
Marital status	Single	23	1.54	0.61	1.69	0.47	3.03	1.16	1.86	*p* = 0.966*	
	Formal relationship	49	1.52	0.62	1.34	0.34	3.12	1.12	1.97		
	Partnership relationship	20	1.60	0.55	1.48	1.00	3.19	1.18	1.95		
	Widowed	8	1.72	0.92	1.62	0.75	3.22	0.87	2.23		
Education	Medical high school	12	1.31	0.33	1.27	0.84	1.94	1.12	1.46	*p* = 0.151*	
	Medical school	8	1.93	0.87	1.83	0.34	3.22	1.61	2.38		
	Bachelor of nursing	48	1.60	0.57	1.69	0.59	3.03	1.18	2.00		
	Master of Science in Nursing	30	1.50	0.71	1.42	0.38	3.19	1.00	1.95		
Specialization	No	71	1.58	0.57	1.62	0.34	3.03	1.19	1.97	*p* = 0.294**	
	Yes	29	1.51	0.75	1.28	0.59	3.22	1.00	1.97		
Qualification course	No	60	1.59	0.64	1.62	0.38	3.22	1.12	1.97	*p* = 0.612**	
	Yes	40	1.51	0.60	1.41	0.34	3.19	1.14	1.96		
Specialized course	No	65	1.54	0.53	1.62	0.38	3.12	1.16	1.97	*p* = 0.842**	
	Yes	35	1.59	0.77	1.47	0.34	3.22	1.09	1.97		
Further training course	No	88	1.53	0.62	1.40	0.38	3.22	1.08	1.94	*p* = 0.067**	
	Yes	12	1.79	0.59	1.77	0.34	2.75	1.65	2.10		
Seniority in the profession	0–5 years	45	1.51	0.59	1.38	0.47	3.03	1.06	2.00	*p* = 0.061*	
	6–10 years	19	1.35	0.45	1.28	0.38	2.00	1.08	1.75		
	11–15 years	16	1.95	0.59	1.91	1.00	3.19	1.60	2.15		
	16–20 years	9	1.46	0.54	1.38	0.94	2.75	1.19	1.59		
	21 years and over	11	1.64	0.91	1.72	0.34	3.22	1.08	1.98		
Number of jobs	1 workplace	45	1.52	0.53	1.41	0.47	2.84	1.12	1.94	*p* = 0.12*	
	2 workplaces	34	1.71	0.63	1.67	0.59	3.19	1.22	2.04		
	3 and more workplaces	21	1.39	0.75	1.31	0.34	3.22	0.94	1.72		
Monthly number of overtime hours	More than 100 h	9	1.74	0.79	1.97	0.59	3.03	1.19	2.06	*p* = 0.543*	
	Approximately 75 h	19	1.36	0.59	1.41	0.34	2.25	0.88	1.80		
	Approx. 50 h	27	1.64	0.55	1.72	0.47	3.12	1.23	1.89		
	Approximately 25 h	32	1.59	0.68	1.31	0.91	3.22	1.08	1.95		
	Not at all	13	1.48	0.56	1.22	0.94	2.75	1.06	2.00		
System of work	Single shift system	5	1.54	1.16	1.00	0.34	3.22	0.94	2.22	*p* = 0.724*	
	Shift system	84	1.57	0.58	1.58	0.47	3.19	1.16	1.95		
	Mixed system	11	1.47	0.74	1.41	0.38	3.03	1.02	1.84		
Number of patients under care while on duty	1–5 patients	12	1.26	0.74	1.09	0.34	3.22	0.99	1.26	*p* = 0.127*	
	6–10 patients	34	1.62	0.55	1.79	0.47	2.53	1.24	2.00		
	11–15 patients	39	1.52	0.62	1.31	0.38	3.12	1.16	1.83		
	16–25 patients	10	1.83	0.77	1.71	1.00	3.19	1.23	2.06		
	26 or more patients	5	1.61	0.48	1.72	1.09	2.12	1.12	2.00		
Participation in training on preventing professional burnout	Yes– A	24	1.54	0.60	1.59	0.34	3.00	1.00	1.98	*p* = 0.028*** C > B	
	No– B	55	1.43	0.56	1.28	0.38	3.03	1.08	1.79		
	Do not remember– C	21	1.91	0.69	1.81	0.88	3.22	1.31	2.12		
Ward	Conservative– A	31	1.81	0.50	1.81	1.06	3.00	1.44	2.03	*P* = 0.021*** A > B,E, C	
	Surgical– B	30	1.55	0.63	1.33	0.38	3.19	1.16	1.91		
	Pediatric– C	12	1.30	0.75	1.11	0.47	3.03	0.65	1.79		
	Pediatric surgery– D	5	1.51	0.62	1.41	0.84	2.22	1.00	2.06		
	ICU– E	20	1.42	0.63	1.30	0.34	3.22	0.99	1.82		

***Notes:*** p*– Kruskal-Wallis test, p**– Mann-Whitney test; p***– Kruskal-Wallis test + post-hoc analysis (Dunn’s test)

The BERNCA-R significantly (p˂0.05) and positively correlates with work disengagement. Therefore, the higher the level of work disengagement, the higher the degree of care rationing (Table 7).

**Table 7:** Results of correlation analysis of BERNCA-R with OLBI

OLBI	BERNCA-R
	Spearman’s correlation coefficient
Exhaustion	*r* = 0.196, *p* = 0.051
Disengagement	*r* = 0.331, *p* = 0.001*

***Abbreviations***: OLBI: the Oldenburg Burnout Inventory, BERNCA-R: the Basel Extent of Rationing of Nursing Care Revised

## Discussion

The main aim of the conducted research was to examine the impact of burnout among nurses on the level of nursing care rationing. In our own study, it was demonstrated that BERNCA-R significantly and positively correlates with emotional disengagement from work. Therefore, the higher the level of disengagement from work, the higher the degree of nursing care rationing. Research conducted by Uchmanowicz et al. [19] indicates a significant correlation between BERNCA-R and MBI (*p* < 0.05). The BERNCA-R result reflected the emotional exhaustion of the examined group. Occupational burnout significantly influences the level of nursing care rationing. In the study by Piotrowska et al. [20] the BERNCA result correlated significantly and negatively with emotional exhaustion, a lack of professional accomplishment, and the overall MBI score. The research suggests that the higher the emotional exhaustion, the higher the level of nursing care rationing.

In our own study, the average total score of BERNCA-R was 1.56 points (SD = 0.62). It can be concluded that the frequency of nursing care rationing among respondents falls between “never” and “rarely.” The most frequently rationed activities by respondents include assisting patients with limited/difficult mobility or immobilized in movement, observing confused patients, requiring their immobilization, activities related to the oral hygiene of patients, activities related to the dental hygiene of patients, and changing the position of patients with limited/difficult mobility or immobilized. Similar results were obtained by Fabich [21] (1.64 ± 0.88). However, in the mentioned study, different frequently rationed activities were identified. These included checking the patient’s condition as precisely as required, talking to the patient or their family, checking the patient’s condition as precisely as prescribed by the doctor, familiarizing oneself with the situation of individual patients and care plans at the beginning of the shift, and assessing the needs of newly admitted patients [21]. In the study conducted by Schubert et al. [13] involving nurses working in the intensive care unit in Sweden, a much lower score was obtained for the entire scale (0.77 ± 0.52). The much lower level of care rationing in intensive care units may result from the specificity of the ward and the patients.

On the other hand, in the study by Baszkiewicz [22], which assessed nursing care rationing in a pediatric hematology ward, the BERNCA-R score was 2.47 points (SD = 0.64), and the frequency of care rationing ranged between “sometimes” and “rarely.” A difference can also be observed in the most frequently rationed activities: talking to the patient and their family, activities related to oral hygiene, developing a care plan for the patient, and monitoring the patient’s condition. Therefore, it can be concluded that the level of nursing care rationing varies depending on the care recipient and the place where care is provided.

In the current study, most participants reported a moderate level of work exhaustion and disengagement from work. The average score for occupational burnout in the study by Piotrowska et al. [20] was 49.27 (SD = 19.76). Emotional exhaustion had the most significant impact on occupational burnout (M = 63.56), contributing to a lesser extent was the lack of a sense of professional achievement (M = 47.05), and depersonalization was the least significant factor (M = 37.2). Conversely, in the study by Uchmanowicz et al. [23], the average score for occupational burnout was 38.14 (SD = 22.93). Emotional exhaustion was the primary factor in occupational burnout (M = 44.8), followed by dissatisfaction with personal achievements (M = 40.66), with depersonalization being the least significant (M = 28.95). In the study by Salvarani et al. [24], emergency nurses characterized by mindfulness, emotion regulation, and empathy skills were better able to cope with work-related stress. Furthermore, it was shown that work-related stress negatively affects the quality of life of cardiac nurses [25].

In the current study, sociodemographic characteristics such as gender, age, postgraduate education (postgraduate and other forms), work experience, and the number of workplaces showed no statistically significant correlation with the BERNCA results. The findings in this study align with results from other research. Radosz et al. [26] confirmed the lack of influence of sociodemographic characteristics on the level of care rationing. Similarly, Wagner-Łosieczka et al. [27] did not find a significant impact of sociodemographic characteristics of nursing staff on the level of nursing care rationing.

In addition to factors related to nursing personnel, the environment and working conditions have a significant impact on care rationing. A study conducted by Piotrowska et al. [20] in oncology departments showed an average BERNCA score of 1.55 (SD = 0.15). Thus, the level of care rationing ranged from “never” to “rarely.” According to research by Uchmanowicz et al. [23], the total BERNCA-R score in cardiology departments was 1.38 (SD = 0.62). This result indicates a frequency of care rationing between “never” and “rarely.” Studies conducted by Baszkiewicz [22] presented the BERNCA score in pediatric hematology and oncology departments at 2.47 (SD = 0.64). Consequently, the frequency of care rationing was between “sometimes” and “rarely.” The results presented partially confirm the findings of the current study. In the author’s research, the frequency of care rationing was higher in adult medical and surgical wards than in adult surgical wards, pediatric wards, and intensive care units (ICUs). The level of nursing care rationing in the studies mentioned above was in the range of “never” to “rarely” in both medical and surgical wards for adults. However, the frequency of care rationing in the third study was higher in pediatric wards, ranging from “sometimes” to “rarely”.

An important aspect of the current study was the questions regarding the expectations of nurses in reducing the level of occupational burnout. Nurses primarily expect the employment of more nurses, higher salaries, a better work atmosphere, increased access to support staff, and improved team communication. The results obtained in the current study partially confirm the findings of other studies or reports in the literature. Rosińczuk et al. [28] present nurses’ expectations regarding work-related changes: salary increases, the possibility of free education, and the creation of a more friendly work atmosphere. According to the study by Kędra et al. [29], respondents answering the question about actions to reduce the level of occupational burnout most frequently indicated: salary increases, taking into account their education, improvement of working conditions in every aspect, and an objective assessment of the work performed.

The concept of occupational burnout has been known and studied for a long time; however, many aspects of this issue remain unexplored. Conducting research on occupational burnout and its impact on nursing care rationing is a new research direction that enables prevention of its occurrence and the potentially harmful consequences for both patients and nurses. Studies aimed at measuring the level of occupational burnout among nurses have significant substantive value. Through such research, management personnel can gain information about occupational burnout, its symptoms, and preventive measures. Enhancing the qualifications of nurses reduces the risk of medical errors. Nurses with greater knowledge and qualifications are more inclined to make independent decisions based on current medical knowledge, significantly reducing the frequency of care rationing.

The study has several limitations. Firstly, it was conducted at a single hospital, which may limit the generalizability of the findings to other healthcare settings with diverse organizational cultures and patient populations. Secondly, the cross-sectional design used provides a snapshot of data at one point in time, making it challenging to establish causality between variables or capture changes over time. Thirdly, reliance on self-report measures, including the OLBI and BERNCA-R questionnaires, introduces the potential for response bias, as participants may provide socially desirable responses. Additionally, the sample size of 100 nurses is relatively small, and the geographic scope is limited, potentially affecting the representativeness and generalizability of the findings. Addressing these potential methodological limitations in future research could strengthen the understanding of the relationship between professional burnout and care rationing among nurses.

## Conclusions

A higher level of nursing care rationing is associated with a higher level of work disengagement. Sociodemographic characteristics of the surveyed nurses do not affect the level of nursing care rationing. Nurses working in the conservative ward more often ration nursing care than those in other wards. The level of nursing care rationing in the surveyed group of nurses was low, ranging between “never” and “rarely.” The level of occupational burnout was moderate, both in terms of work exhaustion and disengagement from work. To minimize occupational burnout, nurses primarily expect: the hiring of more nurses, higher salaries, a better work atmosphere, increased access to support staff, and improved team communication.

## Data Availability

The datasets generated and/or analyzed during the present study are available from the corresponding author upon reasonable request.

## Abbreviations

BERNCA-R: The Basel Extent of Rationing of Nursing Care Revised
OLBI: The Oldenburg Burnout Inventory
